# Further evidence that averageness and femininity, rather than symmetry and masculinity, predict facial attractiveness judgments

**DOI:** 10.1038/s41598-025-86974-0

**Published:** 2025-02-14

**Authors:** Pengting Lee, Jingheng Li, Yasaman Rafiee, Benedict C. Jones, Victor K. M. Shiramizu

**Affiliations:** Department of Psychological Sciences and Health, University of Strathclyde, Glasgow, UK

**Keywords:** Evolution, Psychology

## Abstract

Facial attractiveness influences important social outcomes and most studies investigating possible predictors of facial attractiveness have tested for effects of shape symmetry, averageness (i.e., the converse of distinctiveness), and sexual dimorphism (i.e., masculinity–femininity). These studies have typically either tested for these possible effects by experimentally manipulating shape characteristics in faces images or have tested only for bivariate correlations between shape characteristics and attractiveness judgments. However, these two approaches have been criticised for lacking ecological validity and providing little insight into the independent contributions of symmetry, averageness, and sexual dimorphism, respectively. Moreover, the few studies that have investigated the independent contributions of symmetry, averageness, and sexual dimorphism have reported mixed results. Here we measured shape symmetry, averageness, and sexual dimorphism from face images and assessed their independent contribution to attractiveness ratings. Linear mixed effects models showed that facial attractiveness was significantly predicted by averageness in male and female faces and femininity in female faces, but not by masculinity in male faces or symmetry. These results are consistent with other recent work suggesting that averageness and femininity, rather than symmetry and masculinity, predict facial attractiveness.

## Introduction

Facial-attractiveness judgments influence many important social outcomes^1–3^. For example, people prefer to date, mate with, associate with, hire, and vote for individuals with attractive faces^1–3^. Given this link between facial attractiveness and social outcomes, many researchers have attempted to identify the physical characteristics that influence facial-attractiveness judgments. Because they are hypothesized to function as cues of immunity to infectious illnesses and/or increase the efficiency with which the perceptual system can encode and process faces (see Rhodes^3^ for a discussion of these two accounts of facial-attractiveness judgments), many studies have investigated the possible roles that symmetric, average (i.e., prototypical), and/or sexually dimorphic (i.e., masculine/feminine) shape characteristics play in facial-attractiveness judgments. To elaborate, some researchers have suggested that symmetry, averageness, and sexual dimorphism might influence facial attractiveness judgments because individuals possessing these characteristics also have strong immune systems and, consequently, are produce particularly healthy offspring^4^. However, other researchers have suggested that these characteristics might influence facial attractiveness, not because they advertise aspects of underlying physical condition, but simply because faces possessing these characteristics can be processed most efficiently by the perceptual system^3^.

Many studies have reported that more attractive faces have symmetric^5–8^ and average^8–10^ face shapes. Many other studies have reported that more attractive female faces possess feminine face shapes^11,12^. By contrast with these results, evidence that more attractive male faces have masculine face shapes is rather mixed (see Little et al.^2^ and Rhodes^3^ for reviews). For example, while some studies have reported that masculine male faces are perceived to be more attractive than feminine male faces^11^, other studies have reported that feminine male faces are perceived to be more attractive than masculine male faces^12^ or that masculinity–femininity does not have a significant effect on male facial attractiveness^13^. However, researchers have recently highlighted two potentially important problems with much of this research on the roles that symmetric, average, and sexually dimorphic shape characteristics play in facial-attractiveness judgments.

First, many studies investigating the roles that symmetric, average, and sexually dimorphic shape characteristics play in facial-attractiveness judgments have done so by experimentally manipulating these characteristics in face images using computer graphics methods^5,9,11,12^. However, researchers have recently raised concerns about the ecological validity of this method^14–21^. Indeed, several studies have now demonstrated that the large effects on social judgments of faces that are typically seen when this method is used are considerably smaller, and often not significant, when natural (i.e., unmanipulated) face images are rated and shape characteristics objectively measured from the face images^14,15,17,22–24^. This has led some researchers to propose that experimentally manipulating shape characteristics in face images reveals what features participants *can use* to form impressions of others but does not necessarily reveal the features people actually *do use* under more naturalistic viewing condition where faces vary simultaneously on multiple dimensions (for a discussion of this issue, see Satchell et al.^20^).

Second, most studies that have tested for possible relationships between attractiveness ratings of natural (i.e., unmanipulated) faces and measures of shape symmetry, averageness, or sexual dimorphism have done so by testing for bivariate (rather than partial) correlations only (see Rhodes^3^ for a review). This approach may be somewhat limited because it is not instructive about which of these face-shape characteristics independently predict attractiveness. Indeed, some studies have reported that averageness and symmetry^22,25^ and symmetry and sexual dimorphism are positively correlated in face images^23,26–28^.

Perhaps surprisingly, only a small number of studies have directly addressed these two potentially important issues. However, the specific patterns of results reported differ across these studies. Komori et al.^29^ tested for independent relationships between attractiveness ratings of Japanese faces and both averageness and sexual dimorphism, finding that averageness and masculinity (in male faces) and averageness and femininity (in female faces) independently and positively predicted attractiveness ratings. In a follow-up study, Komori et al.^30^ reported that averageness and symmetry independently and positively predicted attractiveness ratings of Japanese male faces and that averageness, but not symmetry, independently and positively predicted attractiveness ratings of Japanese female faces. By contrast with these results, Pavlovič et al.^31^ tested for independent relationships between attractiveness ratings of Vietnamese faces and symmetry, averageness, and sexual dimorphism, reporting a positive relationship between male attractiveness and averageness, a negative relationship between male attractiveness and masculinity, and a positive relationship between female attractiveness and averageness. By contrast, femininity was not significantly correlated with female attractiveness and symmetry was not significantly correlated with facial attractiveness in either sex. However, a different pattern of results was reported by Kleisner et al.^32^ in a study using a set of faces of diverse ethnicities. In this study, Kleisner et al.^32^ reported independent positive relationships between female attractiveness and averageness and femininity, but not symmetry, and independent positive relationships between male attractiveness and averageness, but not masculinity or symmetry. Thus, while each of these studies reported that averageness was positively correlated with facial attractiveness, findings for symmetry and both masculinity (in male faces) and femininity (in female faces) were mixed across studies.

In light of the mixed results described above, the current study tested for independent relationships between attractiveness ratings of white male and female faces and objective measures of asymmetry, distinctiveness (the converse of averageness), and sexual dimorphism (i.e., masculinity/femininity) of face shape. Given the mixed results from previous studies, we made no specific predictions about which particular features will or will not predict attractiveness significantly in this exploratory study.

## Methods

### Stimuli

**Fig. 1:**
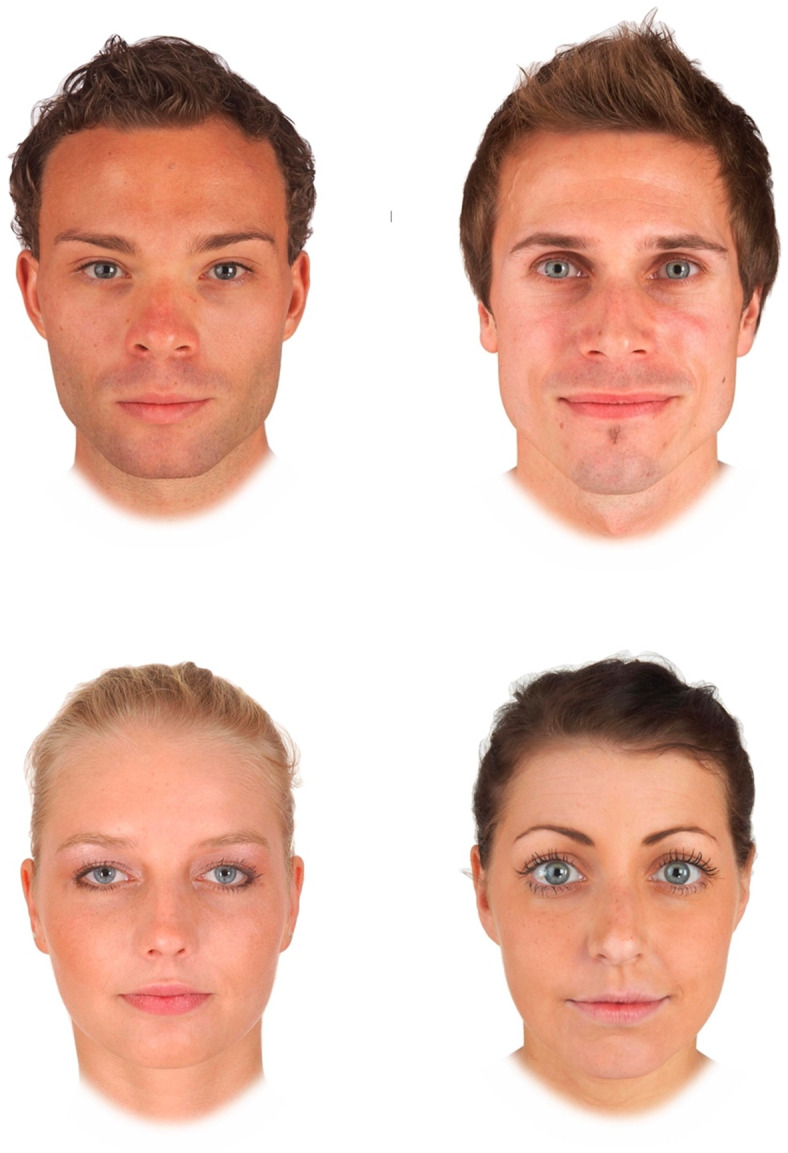
Examples of male (top row) and female (bottom row) face images used in our study.

Stimuli were face images of 50 white men (mean age = 24.2 years, SD = 3.99 years) and 50 white women (mean age = 24.3 years, SD = 4.01 years) that were obtained from an open-access face-image database^33^. Individuals posed front-on to the camera, with direct gaze, and with neutral expressions. Images were standardized on pupil position and clothing was masked prior to rating. Example images are shown in Fig. 1. Images (and the shape templates we used to calculate masculinity, distinctiveness, and asymmetry scores) are publicly available at https://osf.io/a3947/.

### Attractiveness ratings

Two hundred heterosexual men and 200 heterosexual women (mean age = 24.83 years, SD = 5.70 years) were randomly allocated to rate either the 50 male or 50 female face images in an online study using a 7-point scale (1 = much less attractive than average, 7 = much more attractive than average). Trial order was fully randomised. Cronbach’s alphas were high for ratings of both male (Cronbach’s alpha = 0.99) and female (Cronbach’s alpha = 0.99) face images. Cronbach’s alphas were also high for each combination of face sex and rater sex when calculated separately (all Cronbach’s alphas > 0.97). The mean attractiveness rating for male faces was 2.64 (SD = 1.46) and the mean attractiveness rating for female faces was 2.71 (SD = 1.48). Attractiveness ratings were collected using the Experimentum data-collection platform^34^.

### Measuring face-shape sexual dimorphism

Sexual dimorphism of face shape was objectively measured for each of the 50 male and 50 female face images using the facefuns package^35^ in R^36^. This method has been used in many previous studies to assess sexual dimorphism of face shape^14,22,23,37–39^. Shape components were first derived from Principal Component Analysis (PCA) of 132 Procrustes-aligned landmark points (see Holzleitner et al.^38^ for a diagram showing these facial landmarks) on each of the 50 male and 50 female face images. Scores representing sexual dimorphism of face shape were then calculated from each photograph using a vector analysis method^14,22,23,37–39^. This method uses the shape principal components to locate each face on a female-male continuum. The female-male continuum was defined by calculating the average shape information of the 50 female faces and the average shape information of the 50 male faces. Sexual dimorphism scores were then derived by projecting each image onto this female-male vector. Higher scores indicated more masculine face shapes. No scores were more than three standard deviations from the mean (i.e., there were no extreme values).

### Measuring face-shape distinctiveness

Distinctiveness scores were also calculated from each photograph using the facefuns package^35^ in R^36^. This technique has been used to measure face-shape distinctiveness in many previous studies^22,23,37,38^. This method uses the shape principal components described in the previous section of our methods to measure the distance each face lies from the mathematical average shape for the sample of faces of the same sex. That is, the average shape values for the same-sex sample were calculated and, for each image, the Euclidean distance from the average was derived. Higher scores indicate that the face lies a further distance away from the average (i.e., had a more distinctive shape). We measured distinctiveness scores for male and female faces separately in light of evidence that faces are primarily processed relative to sex-specific prototypes^40,41^. No scores were more than three standard deviations from the mean (i.e., there were no extreme values).

### Measuring face-shape asymmetry

Asymmetry scores were also calculated from each photograph using the facefuns package^35^ in R^36^. This technique has been used to measure face-shape asymmetry in many previous studies^22,37,38^. For each image, the landmark template was mirrored, and shape asymmetry measured as the Euclidean distance between original and mirrored templates. Higher scores indicate that the face has greater asymmetry. One extreme value (i.e., one score more than three standard deviations from the mean) was adjusted (i.e., winsorized) to be three standard deviations from the mean prior to further analyses.

## Results

All statistical analyses were carried out using R^36^, with the packages lme4^42^, lmerTest 3.1-3^43^, jtools 2.2.3^44^, and robustHD 0.7.3^45^. Data processing and display used kableExtra 1.3.4^46^ and tidyverse 1.3.1^47^. All data, full outputs, and analysis code are publicly available on the Open Science Framework (https://osf.io/xuz9r/).

**Fig. 2:**
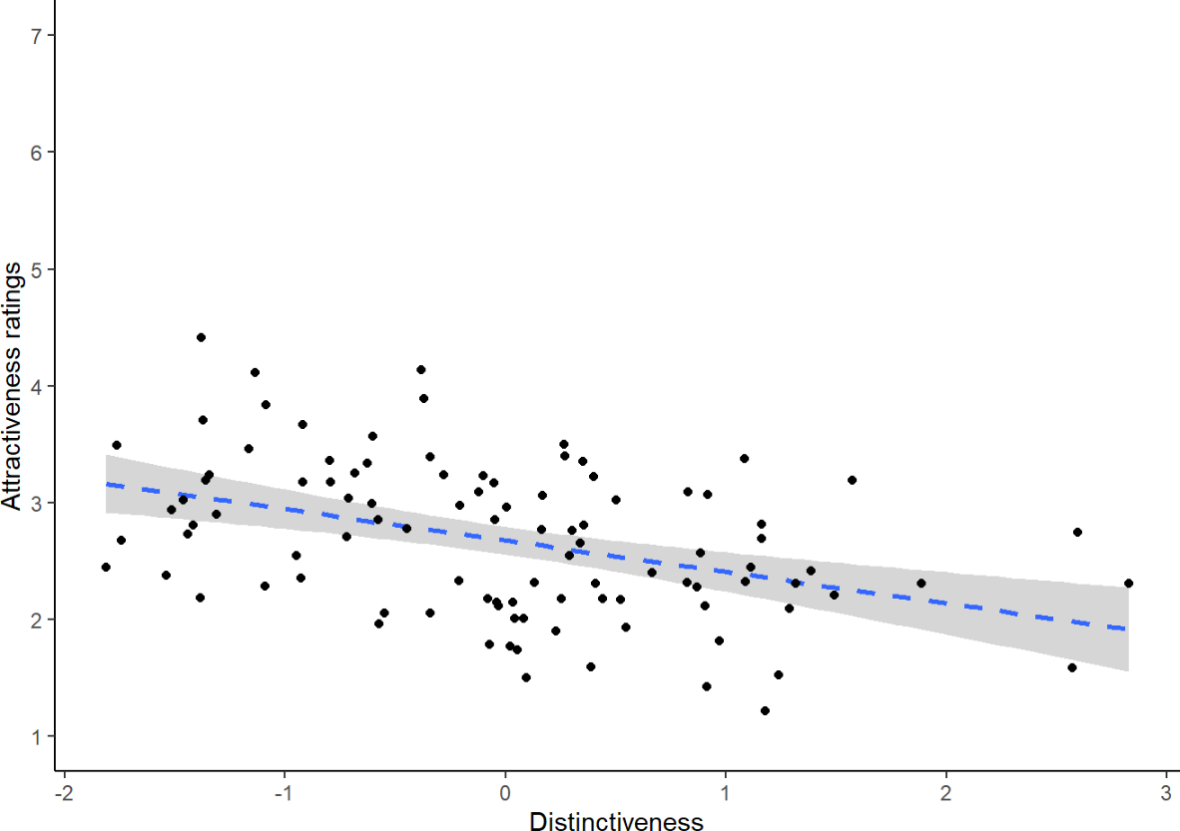
The significant negative effect of face-shape distinctiveness on attractiveness. The shaded area shows the 95% confidence interval.

Attractiveness ratings served as the dependent variable in a linear mixed effects model. The model included main effects of sexual dimorphism scores, distinctiveness scores, asymmetry scores, rater sex (effect coded so that − 0.5 corresponded to male raters and 0.5 corresponded to female raters), and face sex (effect coded so that − 0.5 corresponded to male faces and 0.5 corresponded to female faces) as predictors, as well as all possible two- and three-way interactions (excluding those involving multiple continuous predictors). The model also included by-rater and by-stimulus random intercepts, by-rater random slopes for sexual dimorphism, asymmetry, and distinctiveness (face sex varied between raters), and by-stimulus random slopes for rater sex. Sexual dimorphism, distinctiveness scores, and asymmetry scores were standardised prior to analyses by converting them to z scores (separately for each face sex). Full results for this model are summarised in Table 1. This analysis revealed significant negative main effects of both distinctiveness and sexual dimorphism. By contrast, the effect of asymmetry was not significant. The only significant interaction was the two-way interaction between sexual dimorphism and face sex. The significant effect of distinctiveness is shown in Fig. 2.

**Table 1:** Results of our analysis of attractiveness ratings.

	Estimate	Standard error	t value	df	*p* value
Intercept	2.466	0.108	22.766	131.071	< 0.001
Distinctiveness	− 0.255	0.062	− 4.099	100.778	< 0.001
Face sex	− 0.479	0.217	− 2.212	131.071	0.029
Rater sex	− 0.188	0.085	− 2.227	428.086	0.026
Sexual dimorphism	− 0.278	0.100	− 2.783	101.241	0.006
Asymmetry	− 0.092	0.068	− 1.341	100.493	0.183
Distinctiveness × face sex	0.118	0.124	0.949	100.778	0.345
Distinctiveness × rater sex	− 0.023	0.022	− 1.083	125.376	0.281
Face sex × rater sex	0.239	0.169	1.411	428.086	0.159
Sexual dimorphism × face sex	− 0.53	0.200	− 2.653	101.241	0.009
Sexual dimorphism × rater sex	0.035	0.036	0.970	134.608	0.334
Asymmetry × face sex	0.03	0.137	0.222	100.493	0.825
Asymmetry × rater sex	− 0.007	0.023	− 0.320	117.682	0.750
Distinctiveness × face sex × rater sex	0.001	0.043	− 0.010	125.376	0.992
Sexual dimorphism × face sex × rater sex	0.055	0.072	0.766	134.608	0.445
Asymmetry × face sex × rater sex	0.037	0.047	0.797	117.682	0.427

**Fig. 3:**
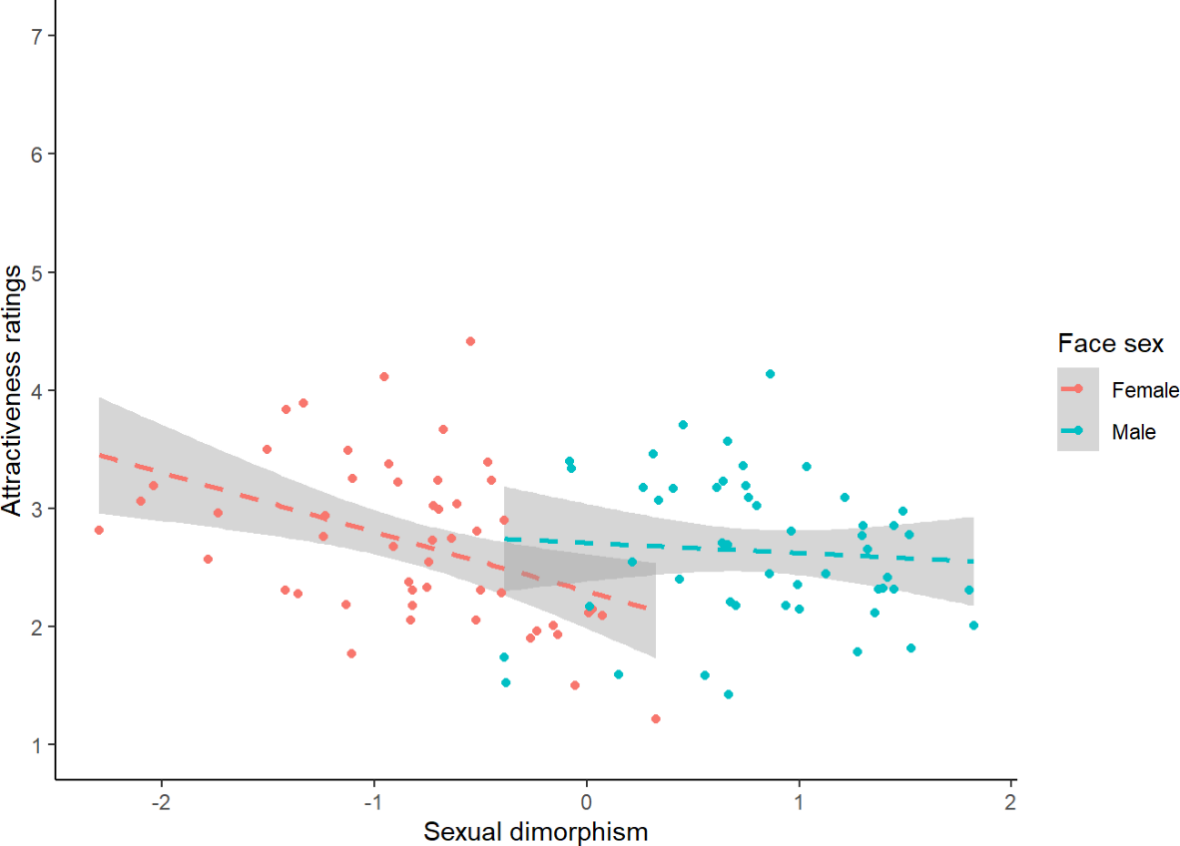
The significant effect of face-shape sexual dimorphism on female attractiveness and non-significant effect of face-shape sexual dimorphism on male attractiveness. The shaded areas show the 95% confidence intervals.

To interpret the significant interaction between sexual dimorphism and face sex, we repeated the analysis described above, this time analysing male and female faces in separate models. For female faces, there was a significant negative effect of sexual dimorphism (estimate = − 0.543, SE = 0.143, t = − 3.803, df = 50.802, *p* < 0.001). By contrast, the effect of sexual dimorphism was not significant for male faces (estimate = − 0.013, SE = 0.138, t = − 0.094, df = 50.145, *p* = 0.926). These results are shown in Fig. 3. Full results for both models are given on the Open Science Framework (https://osf.io/xuz9r/).

We also repeated the initial analysis described above, this time including all possible interactions among predictors in the model. This analysis revealed significant interactions among distinctiveness, rater sex, and asymmetry and among distinctiveness, face sex, rater sex, and sexual dimorphism. Results of these analyses are reported in full at https://osf.io/xuz9r/. Because these high-order interactions were unexpected, we do not discuss them further.

## Discussion

Our analyses of facial-attractiveness ratings showed a significant positive relationship between averageness of face shape and attractiveness ratings of male and female faces (i.e., negative relationships between distinctiveness and facial attractiveness) and a significant positive effect of femininity of face shape and attractiveness ratings of female faces. By contrast, masculinity of face shape was not significantly correlated with male facial attractiveness and symmetry was not significantly correlated with either male or female attractiveness. This pattern of results (significant effects of averageness and female femininity, but not symmetry or male masculinity) is identical to those reported previously by Kleisner et al.^32^. Although other studies testing for independent effects of averageness, symmetry, and sexual dimorphism of face shape on attractiveness ratings also reported positive effects of averageness^29–31^ and female femininity^29^, we did not observe the significant effects of symmetry^30^ or male masculinity^29,31^ that these studies reported. Thus, our findings add to a growing body of research suggesting that averageness is a key shape characteristic for facial attractiveness judgments and that there is little evidence for a significant role of symmetry or male masculinity (see also Kleisner et al.^32^).

There has been considerable debate in the facial-attractiveness literature regarding the extent to which preferences for faces with average shapes are due to a hypothesized positive effect of symmetry on attractiveness ratings^48–50^. However, results from studies in which averageness was experimentally manipulated independently of symmetry suggest that symmetry contributes little to the positive effect of averageness on facial-attractiveness judgments^48–50^. Our finding that averageness, but not symmetry, has an independent positive effect on attractiveness ratings provides further evidence that averageness is attractive independent of the putative effects of symmetry and suggests that symmetry may, in fact, contribute little to the positive effect of averageness on facial attractiveness (see also Kleisner et al.^32^). Although some previous studies have reported that symmetry influences facial attractiveness judgments (see Rhodes^3^ for a meta-analytic review), those studies did not typically control for the positive effects of averageness on attractiveness judgments. This issue is potentially noteworthy, since averageness and symmetry in faces are correlated^22,25^. Indeed, recent work has suggested that effects of averageness mediate effects of symmetry on social judgments of faces^23^. Whether this mediation is due to symmetry and averageness having similar effects on the ease with which faces can be processed by the perceptual system or that both characteristics are associated with similar aspects of physical condition remains an open question. Similarly, whether averageness influences facial attractiveness because average faces can be processed more efficiently or because it advertises physical condition (the two most common explanations for effects of averageness on facial attractiveness, Rhodes^3^) is likely to be an important topic for future research. This issue is also an important direction for future research on the effects of femininity on women’s facial attractiveness.

Our null result for masculinity and male facial attractiveness contrasts with findings from some previous research that found male facial attractiveness was influenced by masculine shape characteristics^11,12,51^. However, studies showing this latter pattern of results typically employed stimuli in which shape characteristics were experimentally manipulated. Several lines of evidence suggest that findings obtained using this type of manipulated stimuli generalise poorly to studies investigating judgments of natural (i.e., unmanipulated) stimuli, as were employed in the current work (see Dong et al.^14^ for a recent review of work on this issue). Nonetheless, while we suggest that differences between the results of the current study and those of previous research on preferences for male facial masculinity may be due to this methodological issue, we also acknowledge that putative effects of contextual factors (e.g., sociocultural factors) on preferences for male facial masculinity^52^ might also contribute to differences in findings across studies.

Although we observed significant relationships between facial attractiveness and face-shape averageness and between shape femininity and female facial attractiveness, these effects were quite weak (see also Kleisner et al.^32^). This issue raises the question of what additional factors might contribute to facial-attractiveness judgments. While most research on facial attractiveness has focussed on investigating possible roles of averageness, sexual dimorphism, and symmetry of face shape, other studies have suggested that surface information (e.g., color and texture information in faces) and cues of adiposity (i.e., facial correlates of body mass index) both may influence facial-attractiveness judgments (see Holzleitner et al.^38^ for a review). Considering these (and other) additional characteristics may improve the predictive power of models of facial attractiveness.

To summarise, much of the research on predictors of facial-attractiveness judgments has focussed on the possible roles played by symmetry, averageness, and sexual dimorphism in shape characteristics. However, relatively few studies have investigated the extent to which these face-shape characteristics independently predict attractiveness and those studies that have done so have reported somewhat mixed results^29–32^. Our finding that facial attractiveness is significantly correlated with averageness in both male and female faces and femininity in female faces, but not masculinity in male faces or symmetry directly replicates the pattern of results reported by Kleisner et al.^32^. Thus, our results present further evidence that averageness and femininity, rather than symmetry and masculinity, predict facial-attractiveness judgments.

## Data Availability

All data, full outputs, and analysis code are publicly available on the Open Science Framework (https://osf.io/xuz9r/).
